# VOCs Profiling and Quality Assessment of Milk Employing Odorant-Binding Proteins-Based Fluorescence Biosensor

**DOI:** 10.3390/ijms27031333

**Published:** 2026-01-29

**Authors:** Cristina Giannattasio, Rosaria Cozzolino, Sabato D’Auria, Angela Pennacchio

**Affiliations:** Istituto di Scienze dell’Alimentazione, Consiglio Nazionale delle Ricerche, Via Roma 64, 83100 Avellino, Italy; cristinagiannattasio93@gmail.com (C.G.); rosaria.cozzolino@cnr.it (R.C.); angela.pennacchio@cnr.it (A.P.)

**Keywords:** odorant-binding protein, fluorescence spectroscopy, gas chromatography-mass spectrometry, biosensors, protein fluorescence, milk quality

## Abstract

The quality of cow’s milk is critical for human nutrition; thus, it is important to develop rapid, sensitive, and cost-effective methods to monitor milk quality. Volatile Organic Compounds (VOCs) from milk are odorant molecules that can be used as key indicators of milk quality, since their presence is influenced by important factors such as animal metabolism, animal diet, and farming practices. In this work, we used the porcine odorant-binding protein (pOBP) and the bovine odorant-binding protein (bOBP) as molecular recognition elements (MREs) of an innovative fluorescence biosensor to detect the presence of odorant molecules in (a) milk produced by intensive livestock farming and (b) milk produced by extensive livestock farming. For biosensors, it is important to use proteins that are stable under operative conditions; therefore, we used fluorescence spectroscopy for a biophysical characterization of the pOBP and of the bOBP at different temperatures. The proposed biosensor employs a system to capture the odorant molecules from milk, which are then transferred to a liquid phase for quantitative and qualitative analyses. The binding of the odorant molecules to the OBPs triggers a Förster Resonance Energy Transfer (FRET) signal, allowing for real-time VOC quantification. The performance of the assays was evaluated by Headspace Solid-Phase Microextraction coupled with Gas Chromatography–Mass Spectrometry (HS-SPME/GC-MS) experiments. The experimental approach used for the development of the biosensor demonstrated high sensitivity and specificity, enabling the differentiation of milk from intensive and extensive farming systems. The results indicate the potential of this method for the real-time monitoring of VOCs in milk samples for food traceability and quality control.

## 1. Introduction

Food traceability is a fundamental aspect of food safety and quality assurance, enabling the monitoring of production chains and ensuring transparency regarding food origins and processing methods. Technologies such as blockchain and artificial intelligence are increasingly used to enhance traceability efficiency, allowing consumers to make informed choices about food products [1]. Milk, rich in proteins, fats, vitamins, and minerals, represents a key nutritional product [2] whose quality and composition are strongly influenced by factors such as diet, farming practices, and environmental conditions [3]. Understanding how these factors may influence the quality and the composition of milk is essential not only for the consumer’s safety but also for optimizing milk production to meet market demands.

VOCs have emerged as crucial biomarkers for milk quality and traceability. These small volatile molecules originate from animal metabolism, feed composition, microbial activity, and environmental conditions [4]. Recent studies have reported that rearing methods and cattle diets greatly affect milk quality, especially focusing on the VOCs’ contribution to the aroma and flavor of milk [3], reflecting the growing consumer interest in sustainable and traceable dairy products and aligning with a broader emphasis on the sensory and compositional qualities of milk [5,6].

Several studies have shown that specific VOCs, such as 1-pentanol, 1-octen-3-ol, and 2-butanone, vary between raw cow’s milk obtained from intensive and extensive farming practices, allowing for the identification of production origins and quality standards [7]. As a result, VOCs profiling has gained attention as a non-invasive and reliable method for milk control and authentication [5,8].

Several analytical techniques can be employed for VOCs detection. Among them, HS-SPME/GC-MS (Headspace Solid-Phase Microextraction coupled with Gas Chromatography–Mass Spectrometry) is the most used method for VOCs sampling and analysis due to its high sensitivity and ability to characterize complex VOCs mixtures [9]. However, due to the complexity of the matrix (fat, water), analysis of VOCs in milk presents some limitations such as the loss of metabolites during the collection process, the low stability of the metabolites, the use of expensive instrumentation and materials (e.g., Tenax tubes, SPME fibers), the presence of trained personnel, and the integration of multiple methodologies (GC-MS, electronic nose) for a comprehensive data collection.

Electronic nose (e-nose) technology offers a promising approach for the rapid detection of milk quality through an array of chemical sensors that mimic the human olfactory system, allowing for the characterization of the VOCs patterns present in milk that can be related to milk freshness or to milk spoilage. The use of E-nose devices is especially valuable for routine freshness assessments and spoilage detection of milk, since the E-nose produces a VOCs “fingerprint” of the milk that is fast to analyze and requires minimal operator training [10]. This fingerprinting provides a practical and non-invasive way to monitor milk quality in both production and storage processes. However, the e-nose has limitations in terms of specificity, as it struggles to distinguish individual compounds within complex mixtures of VOCs, reducing its effectiveness for the identification of specific spoilage markers or the presence of contaminants [11]. Additionally, some environmental variables such as humidity and temperature can affect the analysis of the e-nose, potentially altering the results. Consequently, regular calibration is essential to ensure the accuracy of the results under variable conditions [12].

Biosensors are a rapidly advancing technology for the detection of VOCs in milk, offering a promising alternative for preliminary *on-site* and real-time analysis. These devices integrate MREs such as enzymes, antibodies, or nucleic acid aptamers that specifically bind to target VOCs, triggering a signal that is detectable through transducers like electrochemical, optical, or piezoelectric systems. Biosensors also offer significant advantages in terms of portability and ease of operation, making them particularly well-suited for analysis in the field for milk quality assessments or the rapid detection of the presence of specific contaminants in milk [13].

The need for a rapid, cost-effective, and portable alternative method to analyze VOCs has led to the design of biosensors, which utilize biological recognition elements to detect the presence of specific analytes with high accuracy [14].

Specifically, fluorescence-based biosensors have gained significant interest due to their high sensitivity, rapid response time, and potential for real-time monitoring. One of the most effective fluorescence methodologies used to study biological interactions is the FRET methodology, which relies on the energy transfer between a fluorescence donor molecule and an acceptor molecule, providing an efficient method for the observation of molecular interactions, including VOCs binding [15].

OBPs were used as MREs in the design of the biosensor. OBPs are members of the lipocalin family. They are naturally occurring proteins involved in olfactory perception and capable of binding with small volatile molecules. OBPs have been explored in biosensor applications due to their stability and strong affinity for a wide range of VOCs. In this study, two different OBPs were studied: the pOBP and the bOBP. These two proteins were selected based on their structural properties and binding affinities for the target VOCs associated with the quality of milk. The choice of pOBP as the MRE is strongly supported by its unique structural characteristics and the presence of a single tryptophan residue at position 16, which is pivotal for optimizing a FRET assay. This single tryptophan provides distinct advantages: it emits a specific fluorescence signal, reduces background noise by avoiding overlapping signals from multiple residues, and allows for the precise monitoring of protein–ligand interactions.

The developed biosensor integrates the two OBPs to detect and quantify VOCs in cow’s milk. It provides a novel approach for real-time cow’s milk quality monitoring. For the first time, the performance of the biosensor was evaluated using HS-SPME/GC-MS. The results are presented and discussed.

## 2. Results and Discussion

### 2.1. Docking Results

As reported in the literature, 1-pentanol is a reliable marker of milk produced in extensive farming [7], 2-butanone of milk produced in intensive farming [16], and 1-octen-3-ol of fresh, unpasteurized milk [17]. These VOCs provided a basis for the development of biosensors for milk traceability and quality assessment. This study examined two different OBPs: pOBP, a typical monomeric lipocalin, and bOBP, a de-swapped monomeric derived from bovine. To assess their potential use as MREs, molecular docking studies were conducted to analyze the binding interactions and their affinity for selected VOCs.

Molecular docking analysis was conducted using a rigid receptor approximation, which remains a widely accepted initial strategy for comparative binding studies in OBPs. However, OBPs are not static entities: ligand binding induces local conformational changes in the protein structure, as evidenced by spectroscopic studies on the rat OBP, where binding of the ligand produces detectable structural rearrangements consistent with binding-induced flexibility [18]. Similarly, structural analysis of the mosquito OBP20 reveals significant conformational heterogeneity in the apo state and pronounced structural shifts upon odorant binding, including movements of α-helices that regulate access to the binding pocket, indicating an induced-fit adaptation [19].

Our experimental thermal stability results (increased Tm value upon the ligand binding) are consistent with this behavior, supporting the hypothesis that ligand binding stabilizes a more compact protein conformation. Given these dynamics, the rigid docking models may provide conservative estimates of binding affinity. In addition, since the binding events produce limited receptor flexibility, this would indicate that more favorable ΔG values well-matched with the fluorescence quenching and FRET signals observed are yielded.

#### Direct Docking Analysis

Molecular docking simulation experiments were performed using AutoDock 4.2 to predict the binding affinity of ligands to OBPs. Protein structures were selected based on the high-resolution crystallographic data (bOBP: PDB ID 2HLV, 1.71 Å; pOBP: PDB ID 1DZK, 1.48 Å). A re-docking phase was carried out to optimize simulation parameters, generating 100 conformations of each ligand–protein complex.

The binding affinity was evaluated based on the following:

- Predicted binding free energy (ΔG);
- Cluster population;
- Ligand positioning within the binding site.

**Figure 1:**
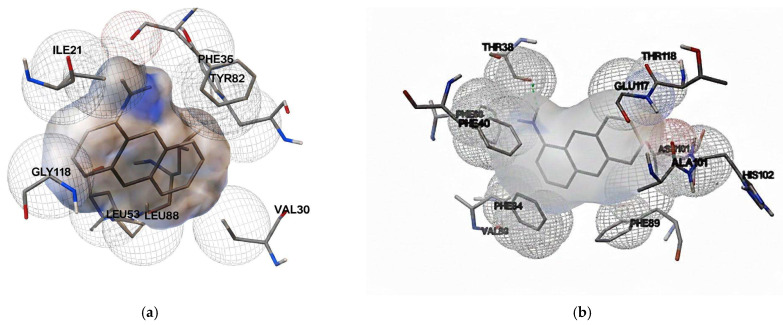
Binding site details in the β-barrel structure of pOBP (**a**) and bOBP (**b**). The amino acid residues involved in interactions with 1-AMA are highlighted.

The lowest energy conformation from the most populated cluster was selected, and interactions were analyzed, revealing predominantly hydrophobic and Van der Waals bonds (Figure 1).

Initial docking simulation experiments were conducted with 1-aminoanthracene (1-AMA), a common fluorophore used in OBP functional assays, to validate ligand binding consistency across OBP variants. Subsequent docking experiments with selected VOCs (1-pentanol, 1-octen-3-ol, and 2-butanone) showed minimal differences in binding energy values between pOBP and bOBP, suggesting similar VOC-binding capabilities. The docking simulation results for additional ligand–protein complexes are provided in the Supplementary Information (Figures S1 and S2).

The predicted binding energy values and the estimated saturation concentrations are reported in Table 1.

### 2.2. Fluorescence Characterization of OBPs

Fluorescence spectroscopy is a widely employed technique to investigate protein conformational changes and ligand-binding interactions. In proteins, the intrinsic fluorescence of tryptophan residues is highly sensitive to changes in the local environment, making it a valuable tool for studying protein folding, stability, and ligand binding. Upon excitation at 295 nm, the indole ring of tryptophan emits fluorescence with a peak near 340 nm. Any perturbations in the microenvironment surrounding the tryptophan residue can alter fluorescence intensity and emission wavelength, providing insights into structural dynamics. A red-shift in fluorescence emission often indicates protein unfolding and subsequent functional loss [20].

FRET was exploited to evaluate protein–ligand interactions. The fluorescent ligand 1-AMA was used as an extrinsic reporter due to its well-characterized ability to bind OBPs with high affinity.

The ability of pOBP and bOBP to bind 1-AMA was assessed through fluorescence titration experiments. Initially, 1-AMA alone exhibited a weak fluorescence emission with a maximum at 537 nm upon excitation at 295 nm. However, upon binding 1-AMA to OBPs, two major spectral changes were observed:

1. A blue-shift in the emission maximum to 481 nm, along with a significant increase in fluorescence intensity.
2. A decrease in the tryptophan fluorescence emission at 340 nm.

These spectral changes indicate the presence of an energy transfer process from the single tryptophan residue (located at position 16 in pOBP and at an equivalent position in bOBP) to the 1-AMA molecules that are intercalated within the protein hydrophobic binding pocket.

**Figure 2:**
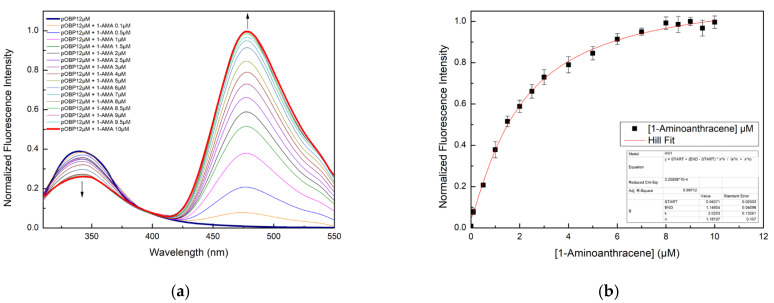
(**a**) Fluorescence emission spectra of pOBP excited at 295 nm in the absence and in the presence of 1-AMA; (**b**) fluorescence emission spectra of pOBP at 481 nm as a function of the concentration of 1-AMA.

**Figure 3:**
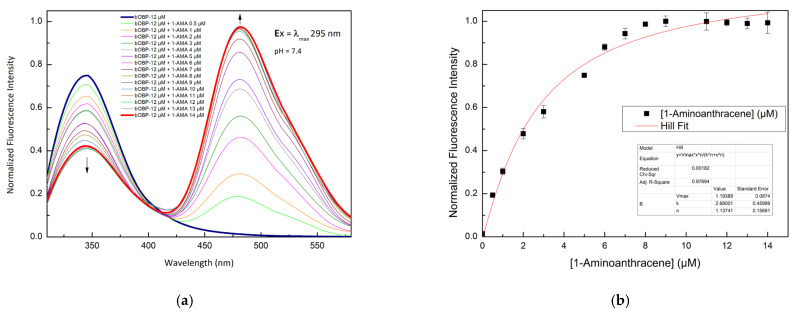
(**a**) Fluorescence emission spectra of bOBP excited at 295 nm in the absence and in the presence of 1-AMA; (**b**) fluorescence emission spectra of bOBP at 481 nm as a function of the concentration of 1-AMA.

The binding affinity values for the 1-AMA binding of pOBP and bOBP were calculated by plotting the fluorescence intensity values as a function of the ligand concentration values, as reported in Figure 2 and Figure 3. pOBP exhibits a higher binding affinity value for 1-AMA compared to bOBP. The measured FRET efficiency values are not directly connected to the binding of VOCs to OBPs, but they indicate that, in the presence of VOCs, there is a displacement of 1-AMA from the protein binding site. In fact, in the OBP–1-AMA complex, a high FRET efficiency is observed, because 1-AMA is well accommodated within the protein hydrophobic pocket at a distance shorter than the Förster radius (R_0_) from the protein tryptophan residues.

Upon the addition of VOCs to the protein solution, a competition between the VOCs and 1-AMA is observed: 1-AMA is displaced from the protein binding site, resulting in a decrease in the FRET signal and a subsequent increase in the protein tryptophan fluorescence emission. This distance-dependent response (E ∝ 1/(1 + (r/R_0_)^6^)) is fully consistent with established fluorescence spectroscopy models, and it is widely applied to study the OBP–ligand systems. Importantly, it is worth highlighting that the ethanol used for VOCs solubilization (below 1% *v*/*v*) does not perturb the OBP structure and the protein binding properties, as demonstrated by the control experiments.

A competitive FRET assay was performed to study the interaction between the OBP and the selected VOCs. In the assay, a solution of OBPs in the presence of 1-AMA was titrated with increasing concentrations of VOCs. The fluorescence emissions at 481 nm (1-AMA bound state) and 340 nm (tryptophan emission) were recorded. A progressive decrease of 1-AMA fluorescence emission and a partial recovery of the protein tryptophan emission were observed. This trend indicates the displacement of 1-AMA from the protein binding site by individual selected VOCs. The variations in the FRET signals, due to competitive binding, were used to quantify the VOC bound to the protein.

**Figure 4:**
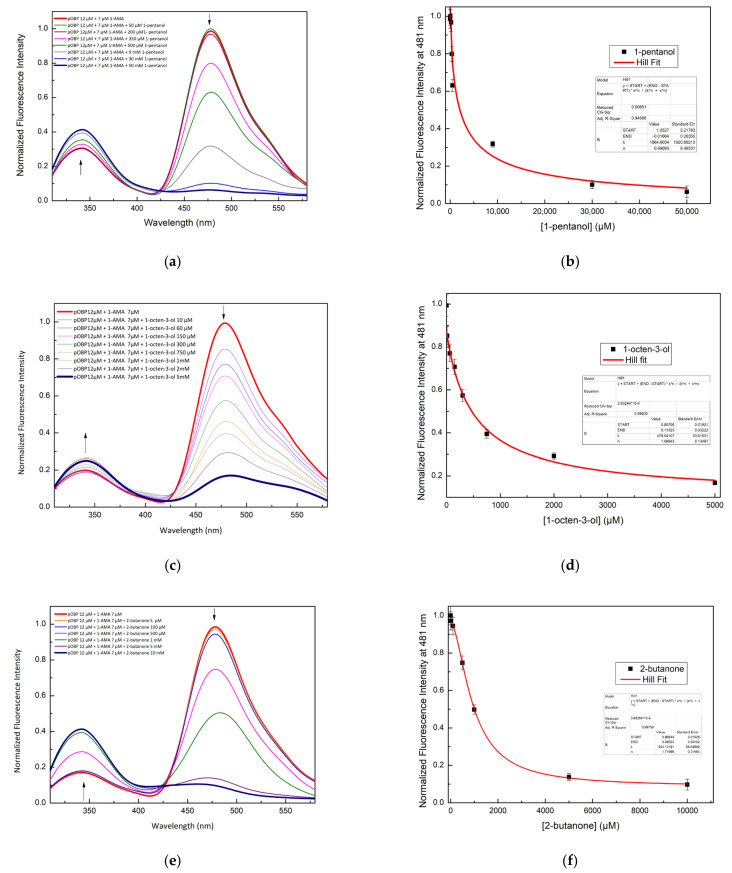
Fluorescence emission spectra of pOBP at increasing concentrations of 1-pentanol (**a**), 1-octen-3-ol (**c**), and 2-butanone (**e**). 1-pentanol (**b**), 1-octen-3-ol (**d**), and 2-butanone (**f**) titration fitting curve. The curves show the decrease in the fluorescence emission at 481 nm of 1-AMA as a function of 1-pentanol, 1-octen-3-ol, and 2-butanone concentrations. The fitting curve obtained by a non-linear function is highlighted in red.

Figure 4 shows the competition process that involves three different VOCs: 1-pentanol (Figure 4a), 1-octen-3-ol (Figure 4c), and 2-butanone (Figure 4e).

**Figure 5:**
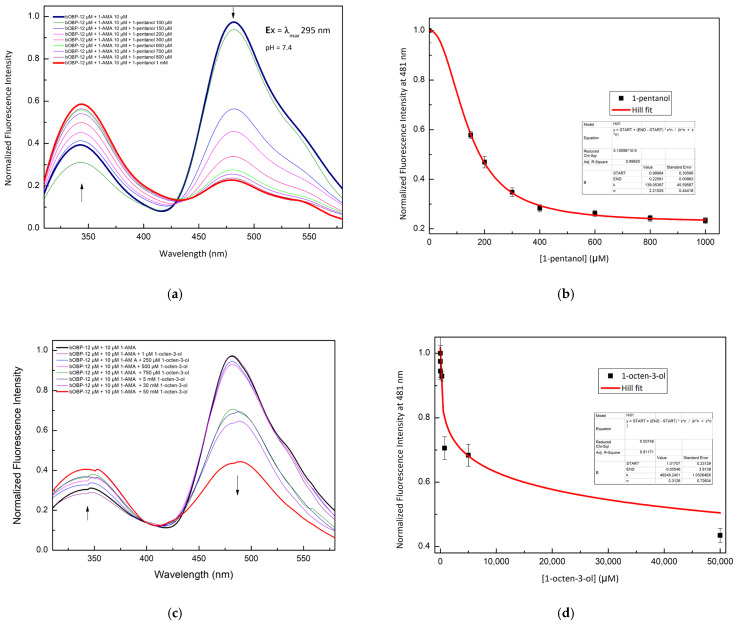
Fluorescence emission spectra of bOBP at increasing concentrations of 1-pentanol (**a**), 1-octen-3-ol (**c**). 1-pentanol (**b**) and 1-octen-3-ol (**d**) titration fitting curve. The curves show the decrease in fluorescence emission intensity of 1-AMA at 481 nm as a function of 1-pentanol and 1-octen-3-ol concentrations. The fitting curve obtained by a non-linear function is highlighted in red.

Figure 5 shows the competition for 1-pentanol (Figure 5a,b) and 1-octen-3-ol (Figure 5c,d) with 1-AMA to the binding site of bOBP.

The constant dissociation values calculated by a non-linear fitting function are reported in Table 2. These results indicate that pOBP and bOBP have different ligand selectivity.

### 2.3. Thermal Stability of pOBP and bOBP

Thermal stability studies were conducted to evaluate the temperature-induced denaturation of the pOBP and bOBP. The experiments aimed to determine the unfolding dynamics of these proteins and assess the influence of VOCs on protein stability. The thermal stabilities of pOBP and bOBP were monitored by fluorescence spectroscopy. The temperature-dependent fluorescence emission spectra were recorded in the range 320–410 nm, with excitation at 295 nm to selectively excite tryptophan residues.

**Figure 6:**
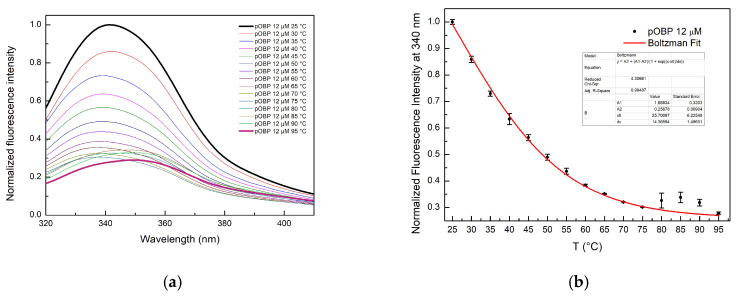
(**a**) Fluorescence emission spectra of pOBP in the temperature range from 25 °C to 95 °C. (**b**) Fitting curve of fluorescence emission intensity at 340 nm as a function of thermal denaturation of pOBP between 25 °C and 95 °C.

**Figure 7:**
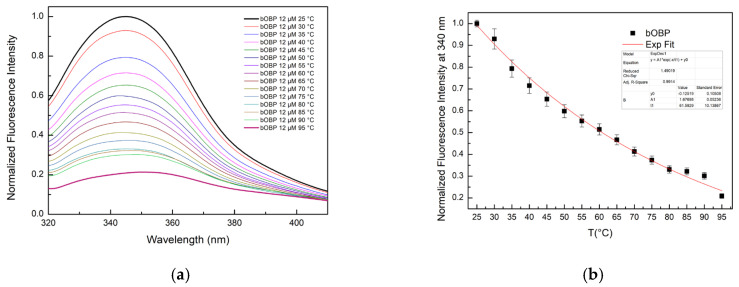
(**a**) Fluorescence emission spectra of bOBP in the temperature range from 25 °C to 95 °C. (**b**) Fitting curve of fluorescence emission intensity at 340 nm.

The fluorescence intensity emission of pOBP progressively decreased as the temperature increased from 25 °C to 95 °C, indicating a protein unfolding process (Figure 6a). This decrease is accompanied by a red-shift in the maximum emission wavelength from 340 nm to 360 nm, a hallmark of protein denaturation due to solvent exposure to tryptophan residues. A similar trend is observed for bOBP. In particular, the denaturation curve revealed a gradual decline in the fluorescence intensity emission, reflecting a loss of the integrity of the tertiary structure (Figure 7a). The fitting curves of the fluorescence emission intensity at 340 nm as a function of the thermal stability of pOBP and bOBP are reported in Figure 6b and Figure 7b, respectively.

According to Staiano et al. (2007) [21], pOBP exhibits high thermal stability with a melting temperature well above the typical physiological range. Specifically, studies using high-sensitivity differential scanning calorimetry (HS-DSC) reported that pOBP unfolds with a transition temperature of approximately 69 °C (≈342 K) at neutral pH values. This indicates the presence of a very stable β-barrel fold in the protein structure that is stable in high temperatures.

The sigmoidal shape of the fluorescence decay curves suggests a cooperative unfolding process. Boltzmann fitting was applied to describe the thermal denaturation behavior and determine the melting temperature (Tm) values, defined as the midpoint of the transition between the folded and unfolded states. The calculated Tm values were 62 ± 0.4 °C for pOBP (Figure 6b) and 54 ± 0.5 °C for bOBP (Figure 7b).

Conversely, the thermal denaturation results of bOBP revealed a more progressive unfolding process. Unlike proteins exhibiting highly cooperative transitions, which typically manifest as a distinct sigmoidal curve, bOBP displayed an exponential decline in fluorescence. This observation points towards a non-cooperative unfolding mechanism, involving the population of several intermediate states (Figure 7b). Such behavior suggests that the bOBP structure is composed of regions possessing differential stabilities, resulting in a stepwise and cumulative loss of its native conformation across the investigated temperature spectrum.

The thermal denaturation results of pOBP and bOBP display different unfolding behaviors. The pOBP denaturation profile exhibits a steep, highly cooperative sigmoidal transition, consistent with a rigid β-barrel stabilized by a dense network of interstrand hydrogen bonds and salt bridges. In contrast, bOBP denaturation profile shows a broader transition, suggesting lower cooperativity and the presence of flexible regions, likely associated with variations in loop length, barrel curvature, or local packing density.

#### 2.3.1. Effect of VOC Binding on Thermal Stability of pOBP and bOBP

To assess whether VOCs contribute to protein stabilization, thermal denaturation experiments were repeated in the presence of saturating concentrations of selected VOCs: 1-pentanol (50 mM), 1-octen-3-ol (5 mM), and 2-butanone (10 mM). The results demonstrated that VOC binding significantly stabilized the pOBP, as evidenced by a shift in Tm towards higher temperatures (Figures S3–S5 in Supplementary Materials). Among the tested VOCs, 1-octen-3-ol exhibited the strongest stabilizing effect, resulting in the highest Tm shift (Tm 64 ± 1.45 °C; Figure S4). This suggests that ligand binding restricts protein flexibility, reducing the likelihood of unfolding at elevated temperatures.

For bOBP, a similar protective effect was observed, although the stabilization was less pronounced compared to pOBP. The Tm values of bOBP increased in the presence of the selected VOCs, with the highest stability conferred by 1-pentanol (Figures S6–S8 in Supplementary Materials). The stabilization mechanism probably involves an enhanced hydrophobic interaction, which can promote the formation of a more compact conformation that is able to resist thermal denaturation.

#### 2.3.2. Comparison of the Thermal Denaturation Curves Exhibited by pOBP and bOBP

**Figure 8:**
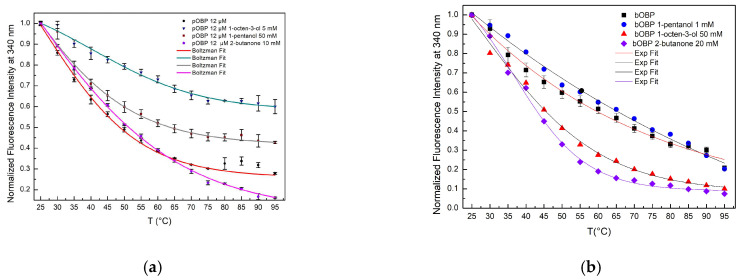
Fitting curves of fluorescence emission intensity at 340 nm as a function of thermal denaturation of pOBP (**a**) and bOBP (**b**) between 25 °C and 95 °C, with saturating concentrations of 1-pentanol, 1-octen-3-ol, and 2-butanone.

Comparative analysis of the denaturation curves of pOBP and bOBP in the absence and in the presence of VOCs points out the role of the ligand in protein structural stabilization (Figure 8a,b). In the absence of VOCs, both pOBP and bOBP exhibit clear thermal denaturation transitions, with a more pronounced cooperative unfolding of pOBP with respect to bOBP. The binding of VOCs to the two OBPs led to a notable increase in protein stability, reducing the overall fluorescence quenching rate and delaying the structural denaturation at elevated temperatures.

The data on the thermal stability experiments show that pOBP becomes more stable in the presence of VOCs. In fact, when the VOCs bind to the pOBP, they interact with key amino acid residues, making the protein more rigid and reducing its flexibility, which helps prevent unfolding. Additionally, VOCs may protect the protein’s hydrophobic core from solvent exposure during heating, preserving protein stability. As a result, the energy required to denature the protein increases, leading to a more gradual unfolding process. In the absence of VOCs, pOBP is more vulnerable to destabilization and unfolding at higher temperatures. As shown in Figure 8a, the largest stabilization occurs in the presence of 1-octen-3-ol, which has the highest affinity for the protein. This stronger interaction contributes significantly to the increased thermal stability observed during the denaturation experiments.

### 2.4. Fluorescence Quenching Studies 

To study the protein conformational changes and accessibility to the tryptophan residues, fluorescence quenching experiments were conducted. According to Lakowicz [20], acrylamide is the most suitable quencher for tryptophan fluorescence. The quenching effect of acrylamide on the intrinsic fluorescence of the protein was analyzed. The study aimed to characterize the accessibility of tryptophan residues, to differentiate between static and dynamic quenching mechanisms, and to assess the effects of the VOCs binding on protein stability [22]. Fluorescence quenching can occur through two primary mechanisms: dynamic (collisional) and static quenching. Dynamic quenching occurs when acrylamide molecules collide with the fluorophore in its excited state, facilitating a non-radiative energy transfer or electron exchange. In contrast, static quenching results from the formation of a non-fluorescent complex between the acrylamide and the protein fluorophore before excitation [23].

To distinguish between these quenching mechanisms, a modified Stern–Volmer equation was applied:

(1) $$\frac{F0}{F}=(1+K_{SV}\;\;\left[Q\right])e^{V\left[Q\right]}$$

where

- F0 and F represent fluorescence intensities in the absence and in the presence of acrylamide, respectively;
- Ksv is the Stern–Volmer constant (indicative of dynamic quenching);
- [Q] is the acrylamide concentration;
- V is the static quenching constant.

#### 2.4.1. Quenching Experiments on pOBP

**Figure 9:**
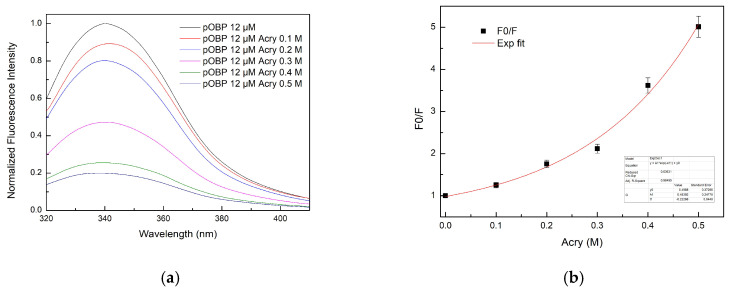
(**a**) Fluorescence emission spectra of pOBP at increasing concentrations of acrylamide (0.1 M, 0.2 M, 0.3 M, 0.4 M, and 0.5 M); (**b**) Stern–Volmer plot of the fluorescence quenching of pOBP as a function of the increasing acrylamide concentration values.

The pOBP fluorescence emission spectra were recorded in the presence of increasing concentrations of acrylamide (Figure 9a). The emission intensity decreased proportionally with the acrylamide concentration increase, indicating an efficient quenching effect. The Stern–Volmer plot (Figure 9b) shows a non-linear trend at higher quencher concentrations (K_SV pOBP = 3 ± 1 M^−1^), suggesting a mixed quenching mechanism.

The presence of VOCs influenced the fluorescence quenching. Figures S9–S11 in Supplementary Materials illustrate the fluorescence emission spectra of pOBP in the presence of saturating concentrations of 1-pentanol, 1-octen-3-ol, 2-butanone, and 1-AMA, respectively, followed by acrylamide titration. The quenching curve transitioned from an exponential to a linear trend, indicating that VOCs binding stabilizes pOBP, reducing the accessibility of tryptophan residues to acrylamide.

#### 2.4.2. Experiments on bOBP Fluorescence Quenching

**Figure 10:**
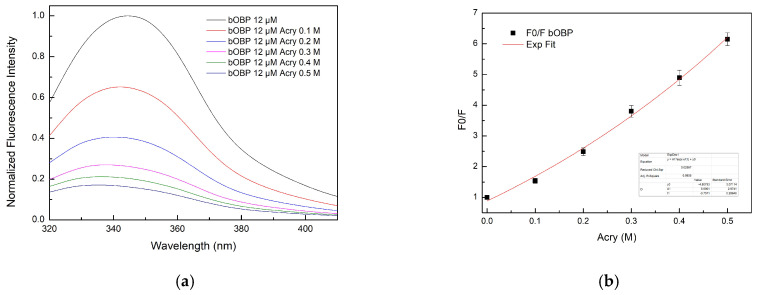
(**a**) Fluorescence emission spectra of bOBP at increasing concentration of acrylamide (0.1 M, 0.2 M, 0.3 M, 0.4 M, and 0.5 M); (**b**) Stern–Volmer plot of fluorescence quenching of the bOBP as a function of increasing acrylamide concentration values.

The fluorescence emission spectrum of bOBP exhibited a broader distribution compared to the pOBP fluorescence emission spectrum, reflecting differences in their structures. Figure 10a presents the fluorescence emission spectra of bOBP titrated with increasing concentrations of acrylamide. A progressive decline in the intensity emission is observed.

The Stern–Volmer plot of bOBP quenching (Figure 10b) reveals an exponential trend (K__SV_ bOBP = 7.5 ± 1 M^−1^), suggesting that the protein structure predominantly underwent dynamic quenching in the absence of VOCs.

Stern–Volmer analysis of pOBP and bOBP reveals a positive deviation from linearity at higher 1-octen-3-ol concentrations, consistent with a mixed quenching mechanism. Dynamic quenching arises from transient collisions of the VOC with fluorophores (Trp or acrylated probe; K__SV_ ~10^5^ M^−1^ for pOBP), while static quenching reflects the stable, complex formation within the hydrophobic pocket. The more flexible entry channel in pOBP favors both frequent collisional encounters and strong static binding, producing a pronounced upward curvature, whereas bOBP shows lower static contributions, likely due to a slightly more rigid pocket. Structurally, dynamic quenching reflects ligand diffusion through the β-barrel entrance, and static quenching occurs when the ligand occupies the core near fluorophores, explaining the mixed behavior observed. The structural integrity at 50 °C is consistent with the rigid scaffold of the lipocalin family. This is supported by previous circular dichroism studies on mammalian OBPs, which show no significant loss of the secondary structure below the onset of the cooperative unfolding transition (T > 55 °C) [21].

#### 2.4.3. Effect of VOCs on Fluorescence Quenching of pOBP and bOBP

The addition of the VOCs to the two proteins modulates the quenching efficiency of both pOBP and bOBP. Figures S12–S14 in Supplementary Materials show the fluorescence spectra and the Stern–Volmer plots for bOBP in the presence of 1-pentanol, 1-octen-3-ol, and 2-butanone. The quenching curve of bOBP shifts towards a more linear response upon VOCs binding, suggesting a stabilization effect that limits fluorophore exposure. At 50 °C, additional quenching experiments were performed to determine possible protein conformational changes. Figures S15 and S16 in Supplementary Materials illustrate the fluorescence spectra and the Stern–Volmer plots for pOBP and bOBP at 50 °C. No significant deviations were observed compared to the experiments performed at 25 °C, suggesting that the proteins retained their structural integrity within this temperature range.

#### 2.4.4. Comparative Analysis of pOBP and bOBP

Distinct quenching behaviors are evident:

- pOBP: Exhibits a predominantly static quenching profile stabilized by VOCs binding.
- bOBP: Displays a dynamic quenching, which indicates higher structural flexibility and exposure of the tryptophan residues.

The linear quenching response of pOBP in the presence of VOCs suggests a tighter binding conformation than bOBP. Conversely, the dynamic quenching of bOBP may indicate multiple binding sites or a more flexible structure, which could enable different interactions with VOCs.

#### 2.4.5. VOC Profiling in Milk Samples

The VOC profile in milk serves as a crucial indicator of farming practices and animal diet. Scientific studies have highlighted distinct differences between milk sourced from intensive and extensive systems [24]. Milk from intensive and semi-intensive systems is characterized by a less diverse VOC profile, where specific ketones, such as acetone and 2-butanone, are predominant. This pattern is thought to reflect a metabolic link to a controlled diet based on total mixed rations (TMR) and silage6. The prevalence of these compounds suggests a standardized and less complex feed intake compared to pasturing animals [5].

In contrast, milk from extensive farming systems exhibits a significantly richer and more complex VOC profile [7]. This greater diversity is attributed to the ingestion of a wide variety of phytochemicals from fresh forage and pastures, which are subsequently metabolized by the animal and transferred to the milk. Key VOCs associated with pasture-fed cows include alcohols, such as 1-pentanol, as well as aromatic hydrocarbons (e.g., toluene) and sulfur compounds (e.g., dimethyl sulfide). The higher number of metabolites belonging to different chemical classes in milk from extensive farming is a direct result of increased dietary diversity [25].

These findings indicate that VOC profiling can be a reliable tool for tracing milk origin and assessing the impact of farming practices and diet on milk quality, confirming that feeding and rearing conditions are determinant factors in the formation of milk’s distinct aroma and flavor.

#### 2.4.6. Binding of Milk VOCs to OBPs

To assess the ability of pOBP to bind VOCs, HS-SPME/GC-MS analyses were carried out on binding assays in the absence and in the presence of the two OBPs. Binding assay experiments were conducted in parallel on samples in the absence (only buffer as negative control) and in the presence of bovine serum albumin (BSA) (used as negative control of protein) or pOBP.

**Figure 11:**
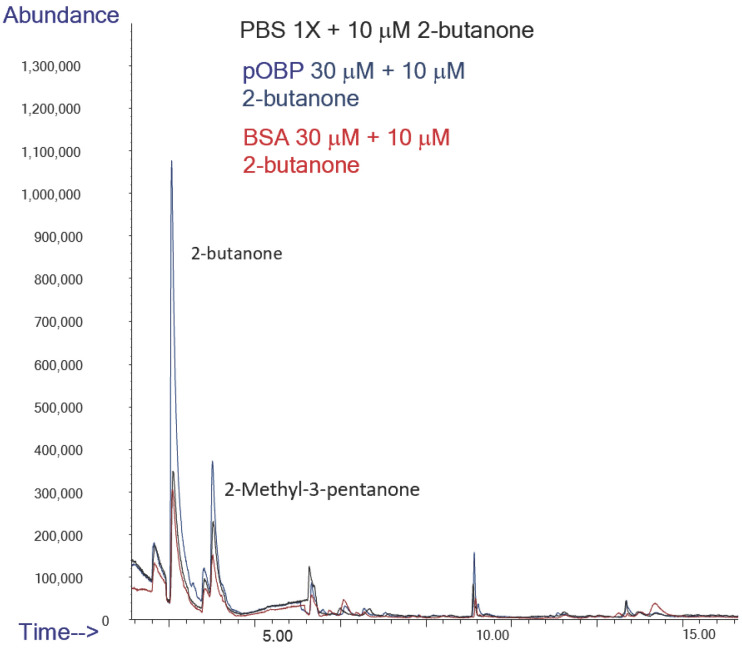
Comparison of Total Ion Chromatograms (TICs) of 2-butanone in the presence of PBS (black line), pOBP (blue line), and BSA (red line), with the inclusion of an internal standard. The TICs highlight the differences in the binding affinities and interaction profiles of 2-butanone with each of the tested proteins, indicating the potential for enhanced sensitivity in detection with pOBP compared to BSA and PBS.

Specifically, the peak area of 2-butanone exhibited a nine-fold increase in the presence of pOBP (blue profile), with respect to the control (black profile) and analysis in the presence of BSA (red profile), confirming a strong affinity between the pOBP and this ketone (Table 3, Figure 11).

**Figure 12:**
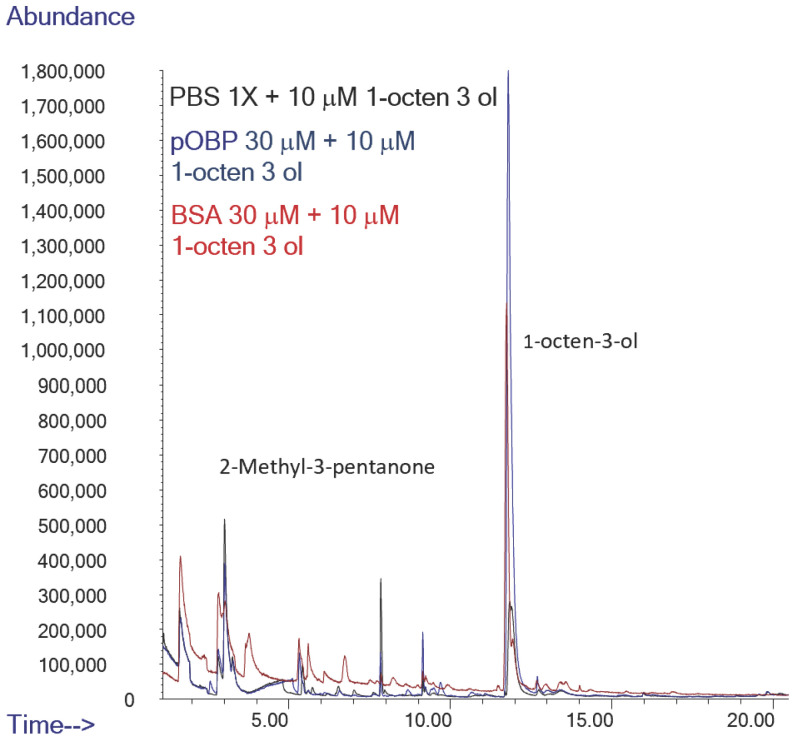
Comparison of Total Ion Chromatograms (TICs) of 1-octen-3-ol in the presence of PBS (black line), pOBP (blue line), and BSA (red line), with the inclusion of an internal standard. The TICs highlight the differences in the binding affinities and interaction profiles of 1-octen-3-ol with each of the tested proteins, indicating the potential for enhanced sensitivity in detection with pOBP compared to BSA and PBS.

The peak corresponding to 1-octen-3-ol, which has a retention time of approximately 12 min in Figure 12, shows an area seven times higher in the presence of pOBP (blue profile), with respect to the control (black line) and to the experiment conducted with BSA (red profile), indicating a discriminative interaction of this metabolite with the pOBP protein (Table 3, Figure 12).

**Figure 13:**
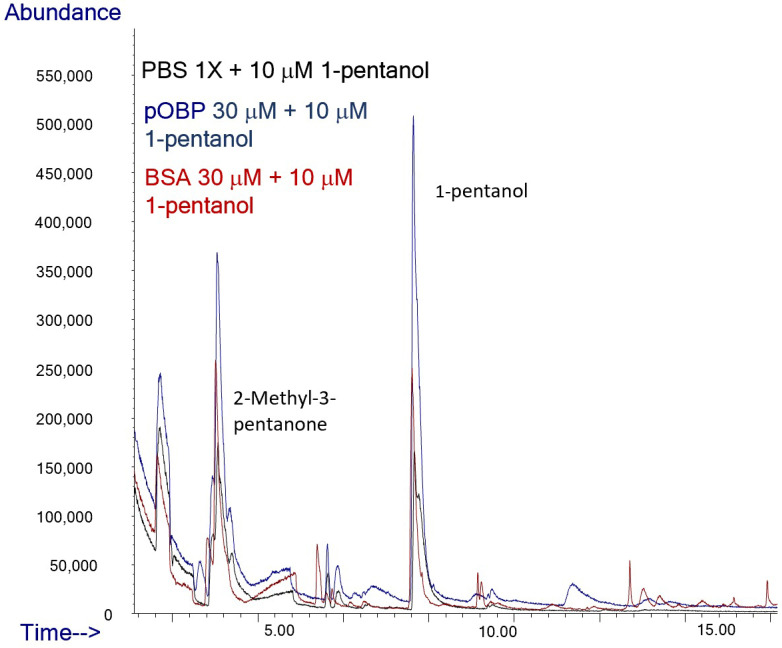
Comparison of Total Ion Chromatograms (TICs) of 1-pentanol in the presence of PBS (black line), pOBP (blue line), and BSA (red line), with the inclusion of an internal standard. The TICs highlight the differences in the binding affinities and interaction profiles of 1-pentanol with each of the tested proteins, indicating the potential for enhanced sensitivity in detection with pOBP compared to BSA and PBS.

The peak area of 1-pentanol shows a 2.5-fold increase in the presence of pOBP compared to BSA (red line) and the control (black line), suggesting a selective interaction of this compound with the pOBP protein (Table 3, Figure 13).

Overall, the results show that the control samples (only PBS) demonstrated no interaction with VOCs, confirming that the detected increase in the peak area is due to the binding of individual volatiles with OBP. Moreover, a direct comparison between pOBP and BSA in the binding assays show the superior selectivity of OBPs for the target VOCs. In fact, when analyzing the peak areas of VOCs in the presence of these proteins, pOBP significantly enhanced the peak areas of each selected volatile (2-butanone, 1-octen-3-ol, and 1-pentanol), while BSA shows a weaker interaction, as expected, indicating a less specific binding mechanism for this protein.

These results highlight the potential of the two OBPs as MREs for VOCs detection, supporting their application in biosensors development.

### 2.5. Impinger System Performance

The impinger system was employed as a volatile compound collection method to introduce selected VOCs (1-pentanol, 1-octen-3-ol, and 2-butanone) into a controlled aqueous environment; this solution containing the VOC was used to monitor the interaction with OBPs using FRET fluorescence assays.

**Figure 14:**
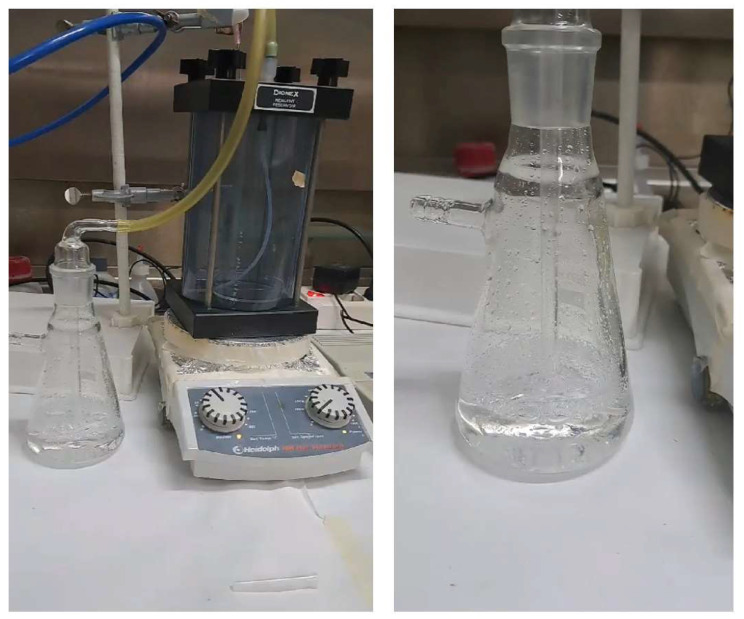
The impinger bubbling mechanism is composed in the Dionex reagent reservoir (California, US) on the magnetic stirrer Heidolph MR Hei-Standard (Schwabach, Germany).

The experimental setup includes a bubbler-based impinger mechanism (Figure 14), wherein a controlled nitrogen gas flow facilitated the volatile metabolite transfer from the gas phase into a Milli-Q water solution. To confirm that bubbling had occurred and that VOCs were present in the solution, competitive FRET assays were conducted. The captured VOC in the liquid was added to a solution containing pOBP saturated with 1-AMA.

**Figure 15:**
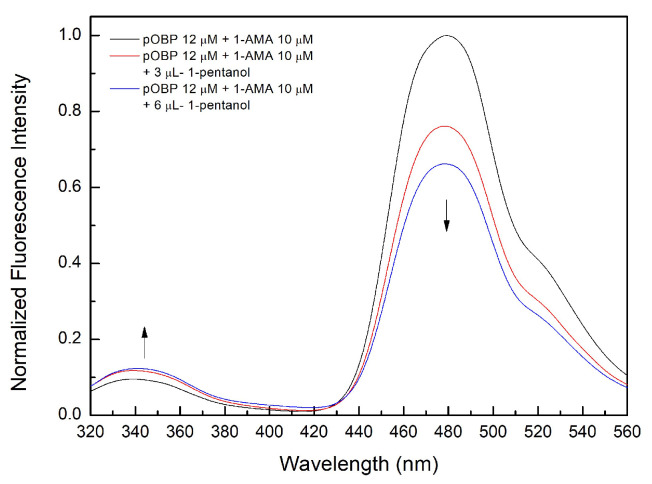
Fluorescence emission spectra of pOBP saturated with 1-AMA at increasing concentrations of 1-pentanol. The addition of 3–6 µL of 1-pentanol determines a decrease in the peak at 481 nm and an increase in the peak at 340 nm.

The observed fluorescence variations after the addition of 3.0 and 6.0 µL of 1-pentanol (Figure 15) support the hypothesis that VOC binding induces structural changes in the pOBP. The concentration of 1-pentanol in the aqueous solution after bubbling was estimated at 2 mM ± 0.4, in line with the expected partitioning behavior of alcohol-based VOCs. The introduction of VOCs into the aqueous phase resulted in a measurable decrease in fluorescence intensity at 481 nm, indicative of pOBP–ligand interactions.

**Figure 16:**
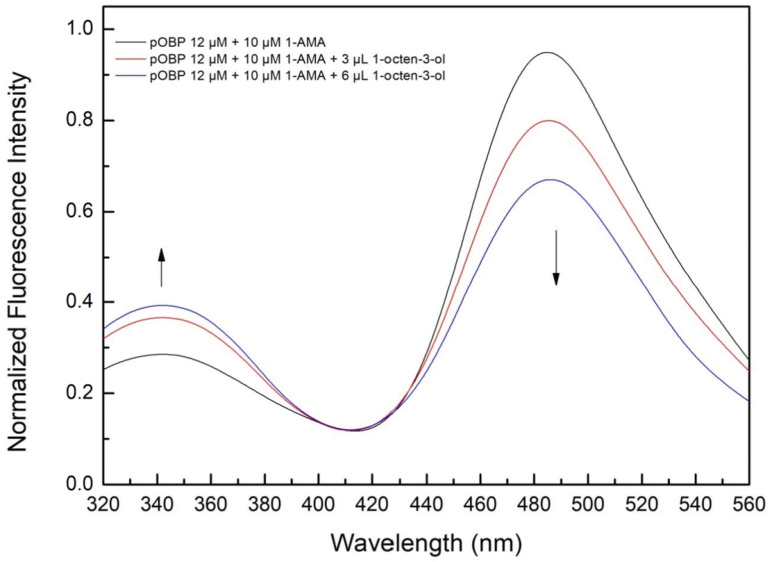
Fluorescence emission spectra of pOBP saturated with 1-AMA at increasing concentrations of 1-octen-3-ol. The addition of 3–6 µL of 1-octen-3-ol determines a decrease in the peak at 481 nm and an increase in the peak at 340 nm.

A similar experiment conducted with 1-octen-3-ol (Figure 16) showed a corresponding fluorescence decrease at 481 nm, confirming its successful capture. The estimated concentration in the aqueous solution was 1 mM ± 0.3, a value lower than that of 1-pentanol, probably due to differences in the volatility and solubility of the different compounds.

A constant nitrogen flow rate of 100 mL/min was maintained at atmospheric pressure. Cross-contamination and VOC carryover were prevented by using fresh protein aliquots for each run and purging the inert PTFE lines with pure nitrogen for 15 min between measurements.

The competitive FRET fluorescence experiment demonstrated a strong correlation between the VOC concentration and fluorescence signal reduction. The progressive quenching of the emission peak at 481 nm upon increasing VOC concentration validated the efficiency of the impinger system in VOC detection. The Hill curve analysis confirmed that the FRET-based sensor was able to detect VOCs at micromolar concentrations, with detection thresholds aligning with previous GC-MS results.

The comparison of fluorescence spectra across different VOCs revealed specific binding affinities for pOBP, confirming its role as a MRE for volatile compounds considered to be markers in milk quality assessments.

The results suggest that the impinger system, in combination with pOBP-based biosensors, can offer a rapid and cost-effective approach for VOCs detection in food matrices. The integration of this technique with machine learning-based predictive models could further enhance VOCs monitoring capabilities.

## 3. Materials and Methods

### 3.1. Materials

2-butanone, 1-pentanol, 2-methyl-3-pentanone, 1-octen-3-ol, and 1-AMA were purchased from Sigma-Aldrich (St. Louis, MI, USA). All solutions were prepared using Milli-Q water (Ultrapure Water Systems Sartorius, Göttingen, Germany). All materials used for protein electrophoresis were purchased from Bio-Rad (Hercules, CA, USA). Glutathione Sepharose 4 Fast Flow (GE Healthcare, Chicago, IL, USA) and HisTrap HP (GE Healthcare, Chicago, IL, USA) resins were used to purify the proteins. Water for mass spectrometry was purchased from Carlo Erba (Milan, Italy). All other chemicals were commercial samples of the best available quality.

### 3.2. Molecular Docking Studies

Molecular docking simulations were performed using the open source software MGLTools (http://mgltools.scripps.edu/). The process began with the preparation of protein and ligand structures using AutoDockTools (ADT) 1.5.6. The structures of bOBP (PDB ID: 2HLV) and pOBP (PDB ID: 1DZK) were retrieved from the Protein Data Bank, while ligand structures were sourced from PubChem in SDF format and subsequently converted to PDB format. The protein structures were refined by verifying their integrity and removing heteroatoms associated with co-crystallized ligands and water molecules. Hydrogen atoms, charges, and atom types were then assigned. The torsional degrees of freedom of the ligands were calculated, and partial charges were added, resulting in the generation of two distinct PDBQT files.

Docking simulations were conducted using a grid box of 60 × 60 × 60 points (spacing: 0.375 Å) for bOBP and 58 × 66 × 46 points (spacing: 0.375 Å) for pOBP. The AutoDock Lamarckian genetic algorithm was employed for docking, performing 100 independent runs while treating the protein as rigid and the ligand as flexible. A total of 30,000 binding poses were generated, with a maximum of 2,500,000 energy evaluations. The affinity maps, computed using AutoDockTools (ADT) 1.5.6., AutoGrid, were saved in GLG format to facilitate binding free energy calculations.

### 3.3. Expression and Purification of OPBs

The pOBP was prepared and purified according to Staiano et al. 2007 [21]. The expression and purification of His-Tagged-bOBP were performed according to Marabotti et al. 2008 [26].

### 3.4. Fluorescence Spectroscopy and FRET Assays

Steady-state fluorescence measurements were conducted using an ISS K2 Frequency-Domain Fluorometer (ISS Inc., Champaign, IL, USA) with a 1.0 cm path length quartz cuvette. Fluorescence spectra of bOBP and pOBP samples were recorded, ensuring optical densities below 0.1 OD at 295 nm to prevent inner filter effects. Tryptophan (Trp) residues were excited at 295 nm, and fluorescence emission spectra were collected from 320 nm to 600 nm at 1 nm intervals, with a scan speed of 100 nm/min and excitation and emission slit widths set at 1.0 nm. The lamp power was maintained at 17.5 A. Assays were performed in 500 μL of phosphate saline buffer (PBS) at pH 7.4. The OBPs-VOCs interactions were studied by a fluorescence titration approach. For this purpose, the protein (12 µM, saturated with 1-AMA) was titrated with increasing concentrations of the tested VOC. The measurements were carried out in triplicate, and the obtained experimental data were processed by OriginPro 2021b software.

For the fluorometric competitive assay, the ability of OBPs to bind VOCs and 1-AMA was utilized to develop a FRET-based assay. The competition between VOCs and 1-AMA for binding to the OBP’s active site was studied by monitoring the fluorescence emission decrease consequent to the VOCs displacement of 1-AMA from the OBP’s active site. The VOCs tested included 1-pentanol (1 µM–50 mM), 1-octen-3-ol (1 µM–5 mM), and 2-butanone (1 µM–10 mM), with ethanol solutions added to bOBP and pOBP samples until the fluorescence emission was stabilized. The measurements were carried out in triplicate, and the data were analyzed using the OriginPro 2021b software.

### 3.5. Thermal Stability Studies

To analyze the protein’s thermal stability, studies on temperature-induced denaturation were conducted on the chosen OBPs in the absence and presence of the selected VOCs. The emission spectra were recorded at an excitation wavelength of 295 nm to focus only on the contribution of tryptophan residues with a 12 μM pOBP sample, with an optical density around 0.1 OD at 295 nm.

Fluorescence intensity variation was recorded in the temperature range from 25 to 95 °C and the emission spectra acquired in the wavelength range 320–410 nm. Measurements were performed in triplicate after 10 min of incubation at defined temperatures. The spectra were first recorded for both proteins (bOBP and pOBP) in the absence and presence of odor molecules (1-pentanol 50 mM, 1-octen-3-ol 5 mM, and 2-butanone 10 mM). To analyze the thermal denaturation data, OriginPro 2021b software was used.

### 3.6. Fluorescence Quenching Experiments

To evaluate the solvent accessibility of the OBP’s tryptophan residues, a fluorescence quenching assay was performed using acrylamide. A solution in water of acrylamide at 7 M was prepared, filtered through a 0.22 µm filter to remove particulates, and stored in the dark at 4 °C, as acrylamide is light sensitive. Six different protein–quencher samples were prepared with increasing concentrations of acrylamide (0 M, 1 M, 2 M, 3 M, 4 M, and 5 M) and incubated overnight. Fluorescence emission spectra were acquired using an excitation wavelength of 295 nm and emission range from 310 to 410 nm.

After completing the acrylamide quenching titration, protein–acrylamide samples were prepared with saturating concentrations of the selected VOCs (1-pentanol 50 mM, 1-octen-3-ol 5 mM, and 2-butanone 10 mM), and the acrylamide titration was repeated.

Quenching data were evaluated using the Stern–Volmer equation by plotting F0/F against [Q], where F0 represents the fluorescence intensity without quencher, F is the fluorescence intensity in the presence of quencher, and [Q] denotes the quencher concentration [Equation (2)]:

(2) $$\frac{F_{0}}{F}=1+K_{SV}\;[Q]$$

Quenching assays involving only the protein–quencher complex were also performed at 50 °C, following the previously described method.

### 3.7. HS-SPME/GC-MS Analysis

The HS-SPME/GC-MS technique was used to evaluate the VOCs profiles of samples of raw cow milk and, successively, the ability of purified OBPs to bind to selected VOCs in the gas phase. According to Sacchi et al., 5 mL of milk were introduced in a HS-SPME 20 mL glass vial, with 5 µL of a standard solution of 2-methyl-3-pentanone used as internal standard (IS, 5 mM). Samples were equilibrated for 30 min at 55 °C to release VOCs. Afterwards, the fiber (DVB-Carboxen-PDMS, 2 cm length SUPELCO, Bellefonte, PA, USA) was automatically inserted in the vial for 60 min and then desorbed for 10 min at 250 °C in the injector port of a GC, model 7890A, Agilent (Agilent Technologies, Santa Clara, CA, USA) coupled to a mass spectrometer, model 5975 C (Agilent), where VOCs were thermally released and directly transported to a capillary column HP-Innowax (30 m × 0.25 mm × 0.5 µm Agilent J&W) for analysis. The oven temperature was set as follows: 40 °C for 1 min, ramped to 160 °C at 6 °C min^−1^, then, after 1 min, increased 10 °C min^−1^ to 210 °C, and finally ramped to 230 °C at 15 °C min^−1^, and kept there for 5 min. The temperatures of the ion source and the quadrupole were 230 °C and 150 °C, respectively, and helium at a flow rate of 1.0 mL min^−1^ was the carrier gas. The analyses were carried out using the pulsed spitless mode, and mass spectra were recorded at an ionization energy of 70 eV, with mass selective detector operating in the mass range between 30 and 300 u. For each sample, three replicates were performed.

Linear retention indices (LRIs) were calculated using the same chromatographic conditions by injecting C8–C40 n-alkane series (Supelco, Bellefonte, PA, USA). VOCs were identified by comparing the mass spectra reported in the NIST-2014 and Wiley 7.0 libraries by matching the calculated LRI with the literature data and by valuating their retention times, with respect to those of pure standards, if available. The VOCs concentrations were expressed as the equivalent of the IS (percent ratio of the respective peak area relative to the IS area, RPA%), and areas were taken from the total ion chromatogram (TIC) according to the following equation: (Peak Area of each VOC/Peak Area IS) × 100.

Using the same experimental parameters reported above, HS-SPME/GC-MS analysis was also performed on pOBP samples with and without exposition to the HS of the selected VOCs. To enable pOBP and volatile binding, vials containing 1× PBS or pOBP (30 µM) were placed in sealed glass bottles with aqueous solutions (10 µM) of individual selected volatiles (1-pentanol, 1-octen-3-ol, or 2-butanone) containing 5 µL of IS and incubated overnight at room temperature. The peak areas measured for PBS-only and pOBP-comprising samples were compared for each experiment. To confirm the binding specificity of each volatile, the same procedure was conducted, replacing pOBP with BSA as a negative control.

### 3.8. Impinger System for VOC Capture

The competitive FRET assay was repeated using the same method reported in paragraph 3, but with the addition of a bubbler. In brief, 2.5 mL of pure solutions of the three selected VOCs were placed in a glass Petri dish, which was positioned inside a sealed container. A constant flow of nitrogen gas was passed through the container to maintain a controlled atmosphere. The apparatus was placed on a heating plate set to 100 °C to facilitate the phase transition of the substances from liquid to gas. The resulting vapors were directed into bubblers containing 100 mL of Milli-Q water and bubbled for two hours. The Milli-Q water saturated with the VOC vapors was then used for the competitive FRET assay.

## 4. Conclusions

In this study, a fluorescence-based biosensor utilizing the two OBPs for the detection of three different VOCs (1-pentanol, 1-octen-3-ol, and 2-butanone) was developed. The relative amounts of these VOCs are different in milk derived from intensive and extensive farming systems. To our knowledge, the HS-SPME/GC-MS technique was used for the first time to explore the ability of pOBP to bind VOCs. Specifically, the integration of HS-SPME/GC-MS and FRET assays allowed for the validation of the pOBP-VOC interaction, supporting the design of a selective biosensing approach. The method used for biosensor development demonstrated high sensitivity and specificity, enabling the differentiation of milk from intensive and extensive farming systems. Specifically, the results obtained suggest the potential of this sensing approach as a promising alternative for the preliminary on-field evaluations of traceability and quality of milk samples. In addition, the absence of preliminary manipulation steps of the milk samples contributes to the preservation of the original chemical VOC signatures of the food matrices. However, to address the challenges of the dairy sector, additional investigations are needed to improve the sensitivity, selectivity, and robustness of the biosensor to progress from scientific laboratories towards industries and markets.

## Figures and Tables

**Table 1:** Direct docking simulation experiments. (ΔG: Estimated Free Energy of Binding; Ki: Estimated Inhibition Constant; mM: millimolar; and µM: micromolar).

VOC	bOBP		pOBP		Amino Acid Residues Involved in Interactions (bOBP)	Amino Acid Residues Involved in Interactions (pOBP)
	ΔG	Ki	ΔG	Ki		
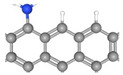 l-Aminoanthracene	−7.39 kcal/mol	3.82 μM	−7.64 kcal/mol	2.50 μM	Thr38-Phe40-Phe54-Val89-Phe89-Ala101-His102-Asn103-Thr116-Glu117	Ile21-Val37-Leu53-Val80-Tyr82-Phe88- Ile100-Asn102-Met114-Gly116-
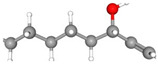 1-octen-3-ol	−4.27 kcal/mol	738.27 μM	−4.84 kcal/mol	284.95 μM	Ile22-Thr36-Thr38-Phe89-Ala101-His102-Asn103-Thr116-Glu117-Phe119	Phe35-Leu53-Val80-Tyr82-Phe88-Asn102-Met114
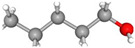 1-pentanol	−2.80 kcal/mol	8.92 μM	−3.11 kcal/mol	5.29 μM	Ile22-Thr36-Phe40-Phe89-Ala101-His102-Asn103-Leu115-Asn117	Ile21-Phe35-Phe88-Thr115-Gly116
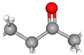 2-butanone	−4.37 kcal/mol	622.37 μM	−4.27 kcal/mol	746.84 μM	Phe54-Phe89-Leu99-Ala101-His102-Phe110-Thr114-Leu115	Ile2l-Phe35-Tyr82-Ile100-Asn102-Met114-Gly116

**Table 2:** pOBP and bOBP dissociation constant (Kd) values for 1-octen-3-ol, 1-pentanol, and 2-butanone.

Ligand	pOBP (Kd ± SD, μM)	bOBP (Kd ± SD, μM)
1-AMA	2.03 ± 0.13	2.68 ± 0.46
1-octen-3-ol	479 ± 53	139 ± 45
1-pentanol	1800 ± 1200	40,000 ± 1000
2-butanone	934 ± 56	/

**Table 3:** HS-SPME/GC-MS semi-quantitative data (%RPA) measured in absence (control) and in presence of BSA or pOBP for 2-butanone, 1-octen-3-ol, and 1-pentanol.

Ligand	PBS 1X	BSA	pOBP
2-butanone	269.8 ± 1.6	276.2 ± 2.7	926.7 ± 10.3
1-octen-3-ol	228.4 ± 14.0	226.7 ± 8.9	708.1 ± 2.7
1-pentanol	229.2 ± 7.0	242.5 ± 5.1	425.0 ± 8.3

## Data Availability

The original contributions presented in this study are included in the article/Supplementary Materials. Further inquiries can be directed to the corresponding author.

## Supplementary Materials

The following supporting information can be downloaded at: https://www.mdpi.com/article/10.3390/ijms27031333/s1.
