# Supplementary material for: VOCs Profiling and Quality Assessment of Milk Employing Odorant-Binding Proteins-Based Fluorescence Biosensor
Source: Int J Mol Sci. 2026 Jan 29;27(3):1333. doi: 10.3390/ijms27031333 (PMC12898745; doi:10.3390/ijms27031333)
Supplement: Supplementary file 1 [file ijms-27-01333-s001.zip › ijms-4024018-supplementary.pdf]

Supplementary Materials

Article

VOCs Profiling and Quality Assessment of Milk Employing Odorant-Binding Proteins-Based Fluorescence Biosensor

Cristina Giannattasio, Rosaria Cozzolino, Sabato D'Auria * and Angela Pennacchio

Istituto di Scienze dell'Alimentazione, Consiglio Nazionale delle Ricerche, Via Roma 64, 83100 Avellino, Italy; cristinagiannattasio93@gmail.com (C.G.); rosaria.cozzolino@cnr.it (R.C.); angela.pennacchio@cnr.it (A.P.)

* Correspondence: sabato.dauria@cnr.it; Tel.: +39-0828299111

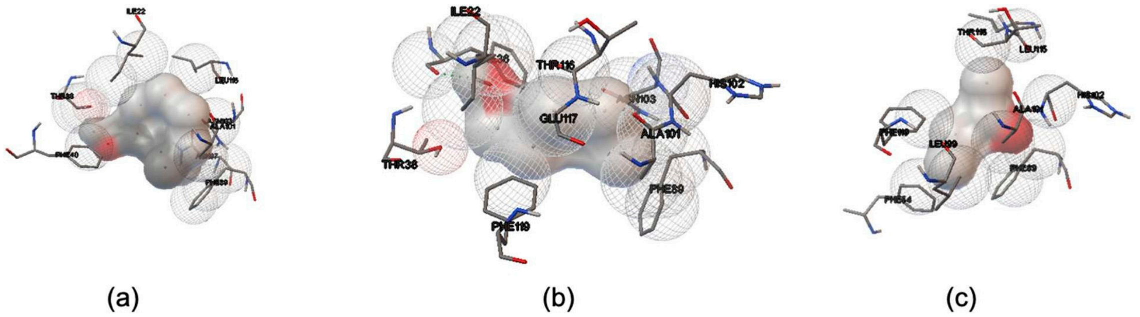

Figure S1. Binding site details in the β-barrel structure of bOBP in complex with **(a)** 1-octen-3-ol, **(b)** 1-pentanol, and **(c)** 2-butanone. The amino acid residues involved in the stabilizing interactions with each ligand are labeled and highlighted.

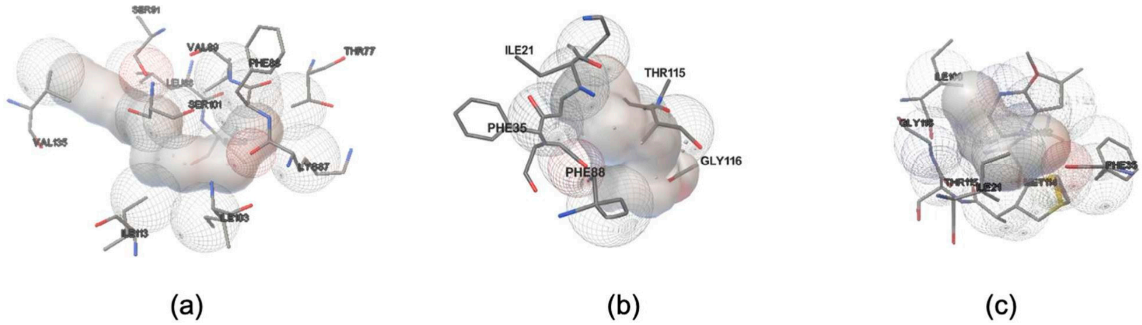

Figure S2. Binding site details in the β-barrel structure of pOBP in complex with **(a)** 1-octen-3-ol, **(b)** 1-pentanol, and **(c)** 2-butanone. The amino acid residues involved in the stabilizing interactions with each ligand are labeled and highlighted.

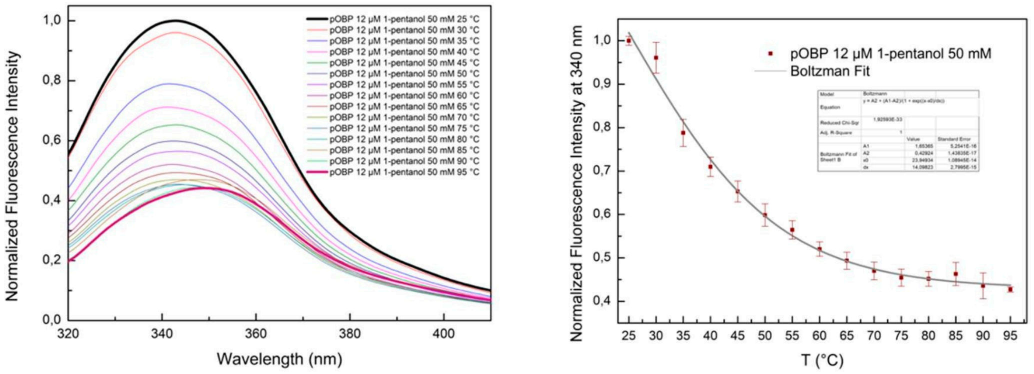

(a) **(b)**

Figure S3. (a) Fluorescence emission spectra of pOBP in the temperature range from 25 °C to 95 °C in the presence of saturating concentrations of 1-pentanol (50mM). **(b)** Fitting curve of fluorescence emission intensity at 340 nm as a function of thermal denaturation of pOBP between 25 °C and 95 °C in the presence of saturating concentrations of 1-pentanol. A shift in Tm towards higher temperatures demonstrates that VOC binding significantly stabilized pOBP.

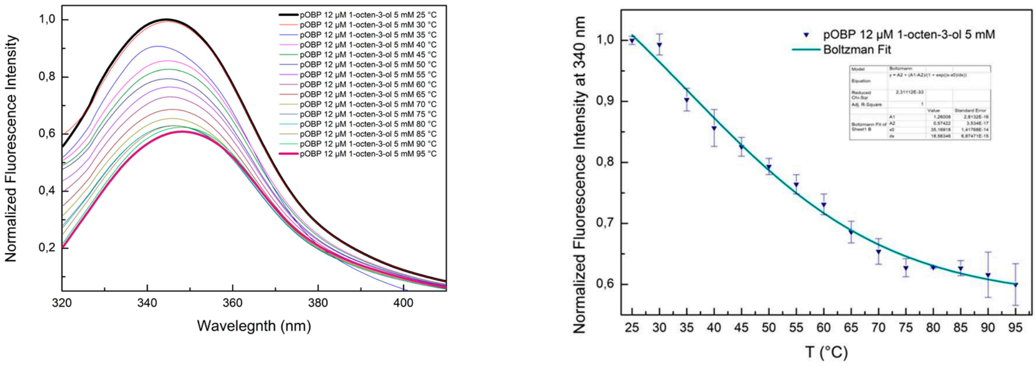

(**a**) (**b**)

Figure S4. (a) Fluorescence emission spectra of pOBP in the temperature range from 25 °C to 95 °C in the presence of saturating concentrations of 1-octen-3-ol (5mM). **(b)** Fitting curve of fluorescence emission intensity at 340 nm as a function of thermal denaturation of pOBP between 25 °C and 95 °C in the presence of saturating concentrations of 1-octen-3-ol. A shift in Tm towards higher temperatures demonstrates that VOC binding significantly stabilized pOBP.

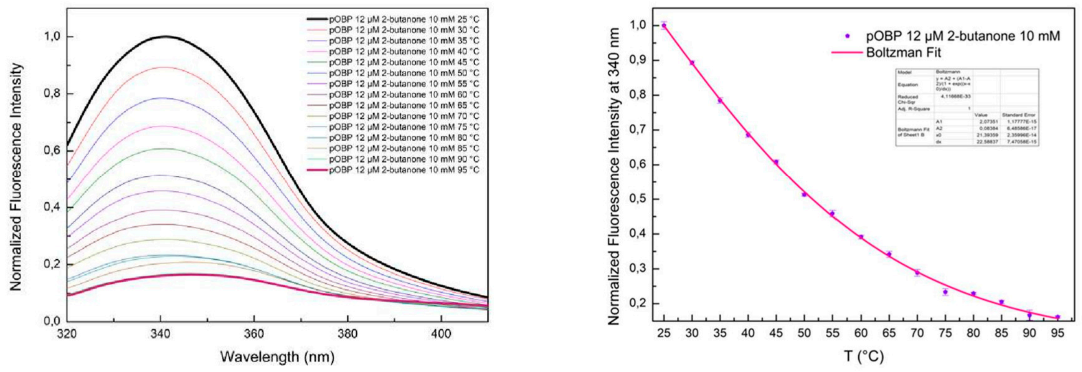

(**a**) (**b**)

Figure S5. (a) Fluorescence emission spectra of pOBP in the temperature range from 25 °C to 95 °C in the presence of saturating concentrations of 2-butanone (10mM). **(b)** Fitting curve of fluorescence emission intensity at 340 nm as a function of thermal denaturation of pOBP between 25 °C and 95 °C in the presence of saturating concentrations of 2-butanone. A shift in Tm towards higher temperatures demonstrates that VOC binding significantly stabilized pOBP.

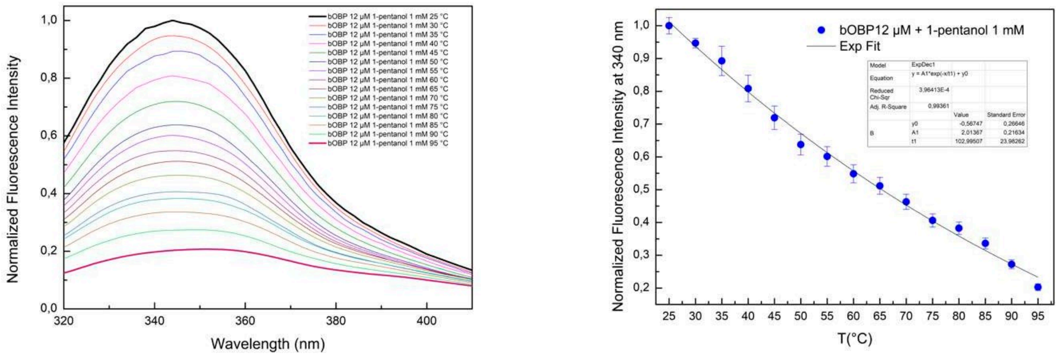

(**a**) (**b**)

Figure S6. (a) Fluorescence emission spectra of bOBP in the temperature range from 25 °C to 95 °C in the presence of saturating concentrations of 1-pentanol (1mM). **(b)** Fitting curve of fluorescence emission intensity at 340 nm as a function of thermal denaturation of bOBP between 25 °C and 95 °C in the presence of saturating concentrations of 1-pentanol. A shift in Tm towards higher temperatures demonstrates that VOC binding significantly stabilized pOBP.

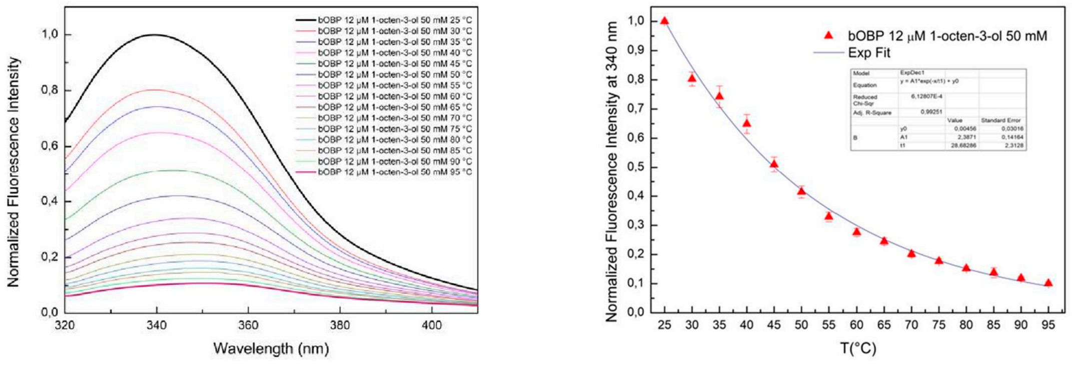

(**a**) (**b**)

Figure S7. (a) Fluorescence emission spectra of bOBP in the temperature range from 25 °C to 95 °C in the presence of saturating concentrations of 1-octen-3-ol (5mM). **(b)** Fitting curve of fluorescence emission intensity at 340 nm as a function of thermal denaturation of bOBP between 25 °C and 95 °C in the presence of saturating concentrations of 1-octen-3-ol. A shift in Tm towards higher temperatures demonstrates that VOC binding significantly stabilized pOBP.

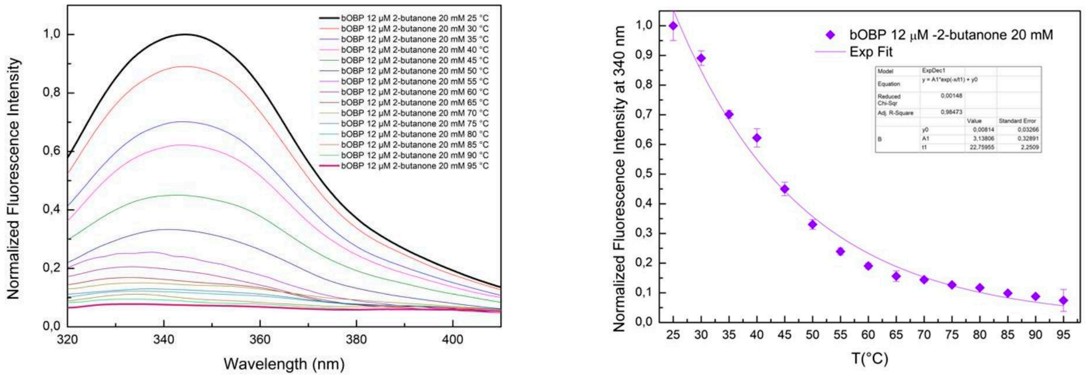

(**a**) (**b**)

Figure S8. (a) Fluorescence emission spectra of bOBP in the temperature range from 25 °C to 95 °C in the presence of saturating concentrations of 2-butanone (10mM). **(b)** Fitting curve of fluorescence emission intensity at 340 nm as a function of thermal denaturation of bOBP between 25 °C and 95 °C in the presence of saturating concentrations of 2-butanone. A shift in Tm towards higher temperatures demonstrates that VOC binding significantly stabilized pOBP.

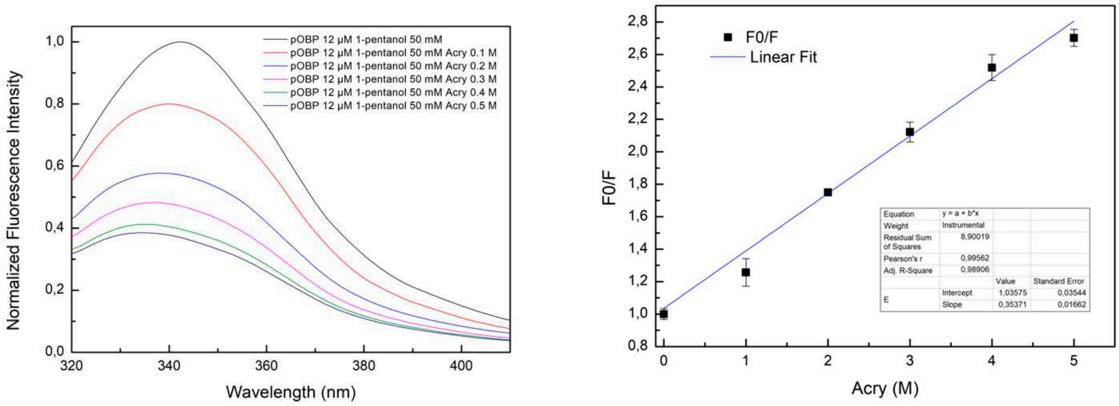

(**a**) (**b**)

Figure S9. (a) Fluorescence emission spectra of pOBP saturated with 50mM 1-pentanol at increasing concentration of acrylamide (0,1 M, 0,2 M, 0,3 M, 0,4 M, 0,5 M). **(b)** Stern-Volmer plots of pOBP saturated with 50 mM 1-pentanol at increasing concentration of acrylamide (0,1 M, 0,2 M, 0,3 M, 0,4 M, 0,5 M). According to Equation 1, the quenching constant Ksv corresponding to the plot slope.

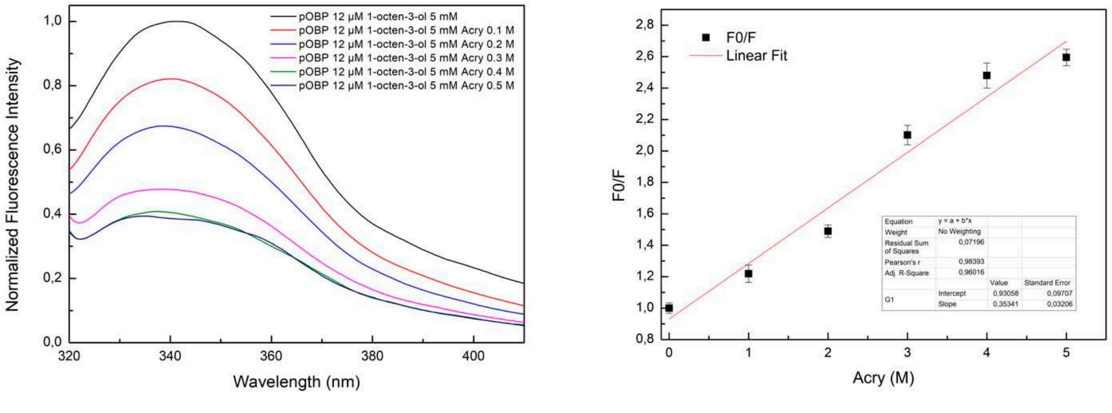

(**a**) (**b**)

Figure S10. (a) Fluorescence emission spectra of pOBP saturated with 5 mM 1-octen-3-ol at increasing concentration of acrylamide (0,1 M, 0,2 M, 0,3 M, 0,4 M, 0,5 M). **(b)** Stern-Volmer plots of pOBP saturated with 5 mM 1-octen-3-ol at increasing concentration of acrylamide (0,1 M, 0,2 M, 0,3 M, 0,4 M, 0,5 M). According to Equation 1, the quenching constant Ksv corresponding to the plot slope.

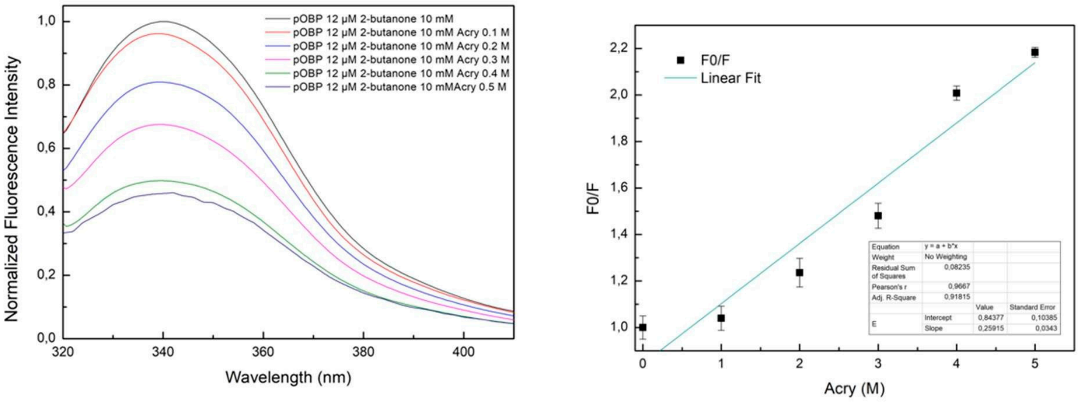

(**a**) (**b**)

Figure S11. (a) Fluorescence emission spectra of pOBP saturated with 10 mM 2-butanone at increasing concentration of acrylamide (0,1 M, 0,2 M, 0,3 M, 0,4 M, 0,5 M). **(b)** Stern-Volmer plots of pOBP saturated with 10 mM 2-butanone at increasing concentration of acrylamide (0,1 M, 0,2 M, 0,3 M, 0,4 M, 0,5 M). According to Equation 1, the quenching constant Ksv corresponding to the plot slope.

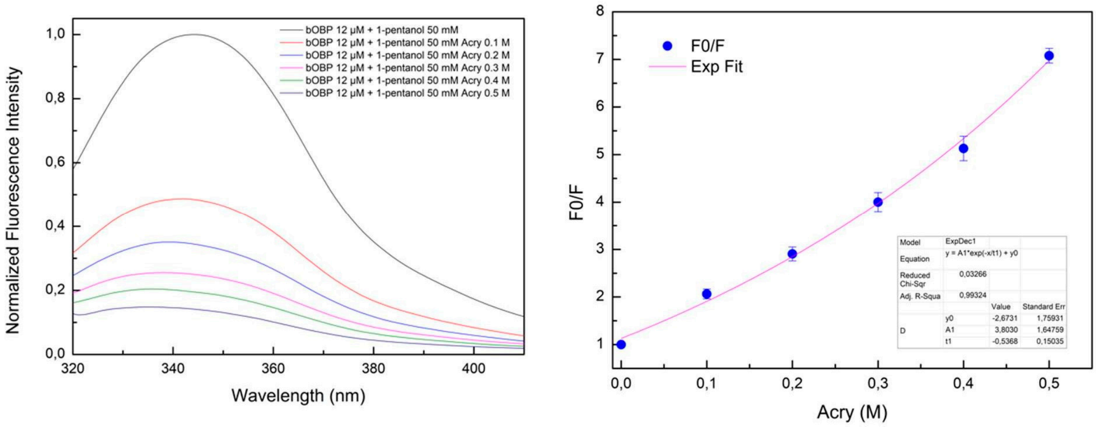

(**a**) (**b**)

Figure S12. (a) Fluorescence emission spectra of bOBP saturated with 50mM 1-pentanol at increasing concentration of acrylamide (0,1 M, 0,2 M, 0,3 M, 0,4 M, 0,5 M). **(b)** Stern-Volmer plots of bOBP saturated with 50 mM 1-pentanol at increasing concentration of acrylamide (0,1 M, 0,2 M, 0,3 M, 0,4 M, 0,5 M). According to Equation 1, the quenching constant Ksv corresponding to the plot slope.

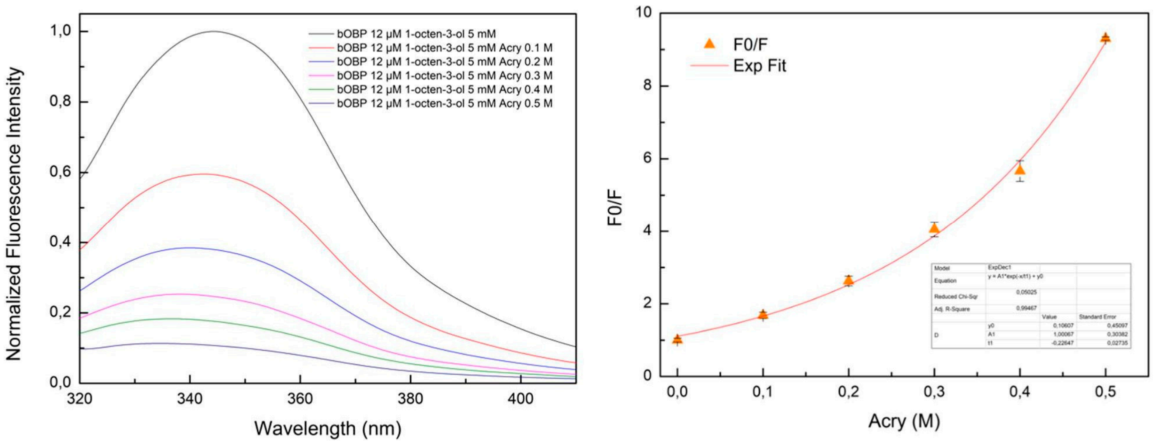

(**a**) (**b**)

Figure S13. (a) Fluorescence emission spectra of bOBP saturated with 5 mM 1-octen-3-ol at increasing concentration of acrylamide (0,1 M, 0,2 M, 0,3 M, 0,4 M, 0,5 M). **(b)** Stern-Volmer plots of bOBP saturated with 5 mM 1-octen-3-ol at increasing concentration of acrylamide (0,1 M, 0,2 M, 0,3 M, 0,4 M, 0,5 M). According to Equation 1, the quenching constant Ksv corresponding to the plot slope.

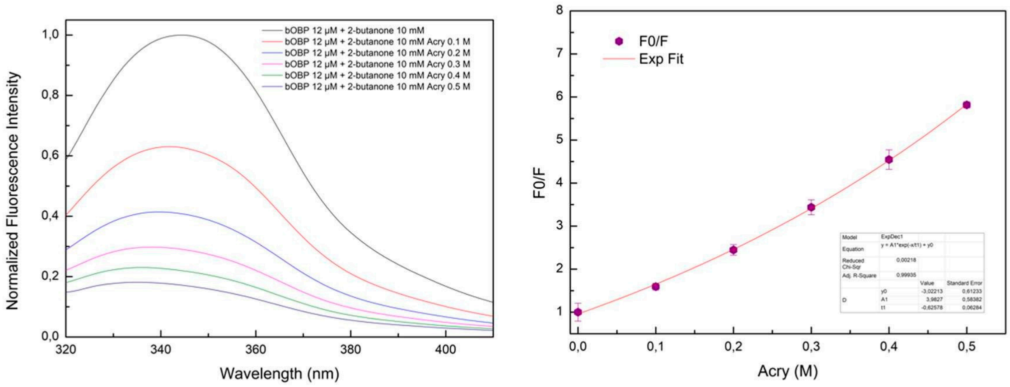

(**a**) (**b**)

Figure S14. (a) Fluorescence emission spectra of bOBP saturated with 10 mM 2-butanone at increasing concentration of acrylamide (0,1 M, 0,2 M, 0,3 M, 0,4 M, 0,5 M). **(b)** Stern-Volmer plots of bOBP saturated with 10 mM 2-butanone at increasing concentration of acrylamide (0,1 M, 0,2 M, 0,3 M, 0,4 M, 0,5 M). According to Equation 1, the quenching constant Ksv corresponding to the plot slope.

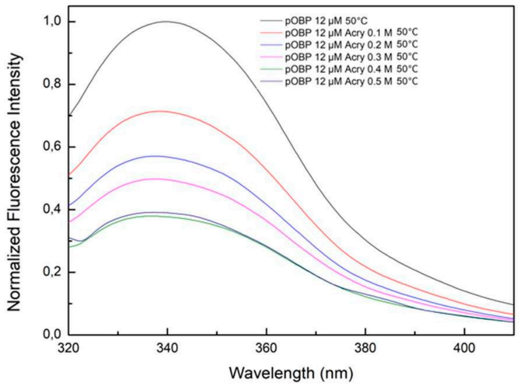

(**a**)

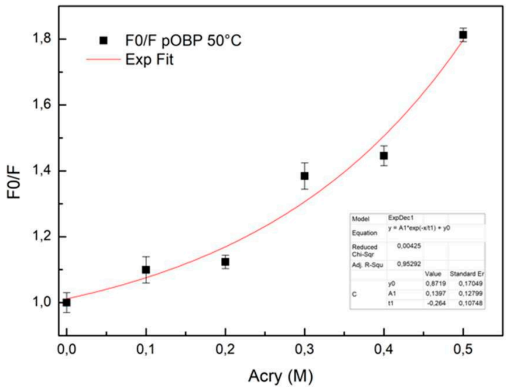

(**b**)

Figure S15. (a) Fluorescence emission spectra of pOBP at 50 °C with increasing concentration of acrylamide (0,1 M, 0,2 M, 0,3 M, 0,4 M, 0,5 M). Fluorescence emission spectra were acquired using an excitation wavelength at 295 nm and emission range from 310 to 410 nm. **(b)** The samples were prepared with 12 μM pOBP and saturating concentrations of the selected VOCs (1-pentanol 50 mM, 1-octen-3-ol 5 mM, and 2-butanone 10 mM).

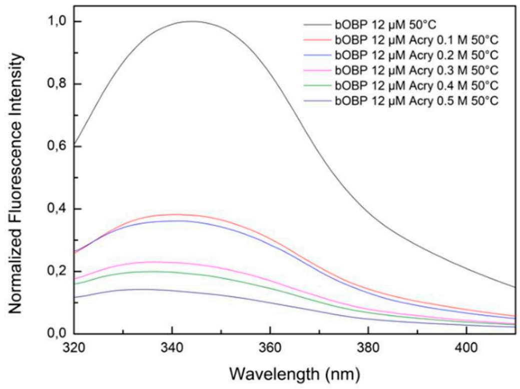

(**a**)

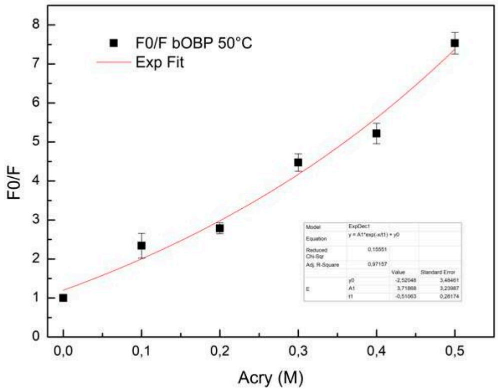

(**b**)

Figure S16. (a) Fluorescence emission spectra of bOBP at 50 °C with increasing concentration of acrylamide (0,1 M, 0,2 M, 0,3 M, 0,4 M, 0,5 M). Fluorescence emission spectra were acquired using an excitation wavelength at 295 nm and emission range from 310 to 410 nm. **(b)** The samples were prepared with 12 μM pOBP and saturating concentrations of the selected VOCs (1-pentanol 50 mM, 1-octen-3-ol 5 mM, and 2-butanone 10 mM).
